# Supplementary material for: Naegleria amoebae seek confinement and crawl persistently through narrow spaces
Source: bioRxiv. 2025 Dec 1:2025.11.30.691445. Preprint. [Version 1] doi: 10.1101/2025.11.30.691445 (PMC12687775; doi:10.1101/2025.11.30.691445)
Supplement: 3 [file NIHPP2025.11.30.691445v1-supplement-3.pdf]

bioRxiv preprint doi: https://doi.org/10.1101/2025.11.30.691445; this version posted December 1, 2025. The copyright holder for this preprint (which was not certified by peer review) is the author/funder, who has granted bioRxiv a license to display the preprint in perpetuity. It is made available under aCC-BY 4.0 International license.

SUPPLEMENTAL FIGURES

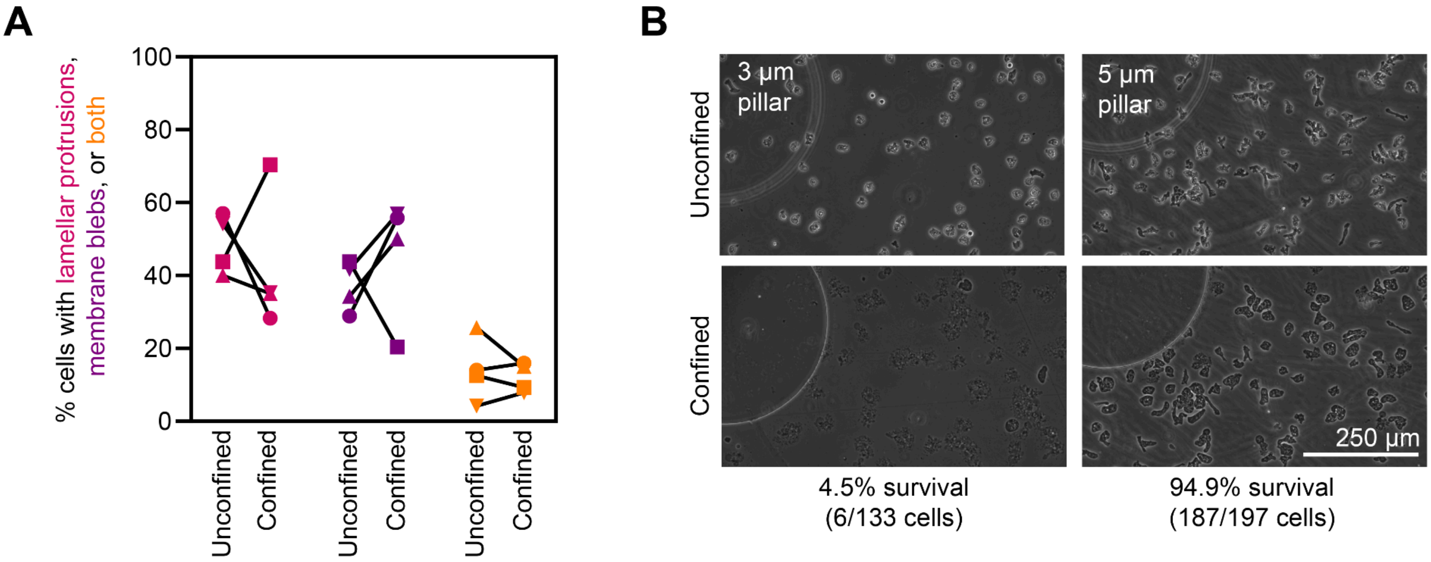

Figure S1. Confinement between coverslips leads to variable phenotypes. (A) The data from individual trials using confinement coverslips which were combined in Fig. 1C are shown by replicate (coordinated by shape). **(B)** 3 µm and 5 µm pillar sizes were tested to determine which size led to optimal confinement between coverslips. Confinement to 3 µm caused the vast majority of cells to burst. Images show the same representative area before and after confinement.

bioRxiv preprint doi: https://doi.org/10.1101/2025.11.30.691445; this version posted December 1, 2025. The copyright holder for this preprint (which was not certified by peer review) is the author/funder, who has granted bioRxiv a license to display the preprint in perpetuity. It is made available under aCC-BY 4.0 International license.

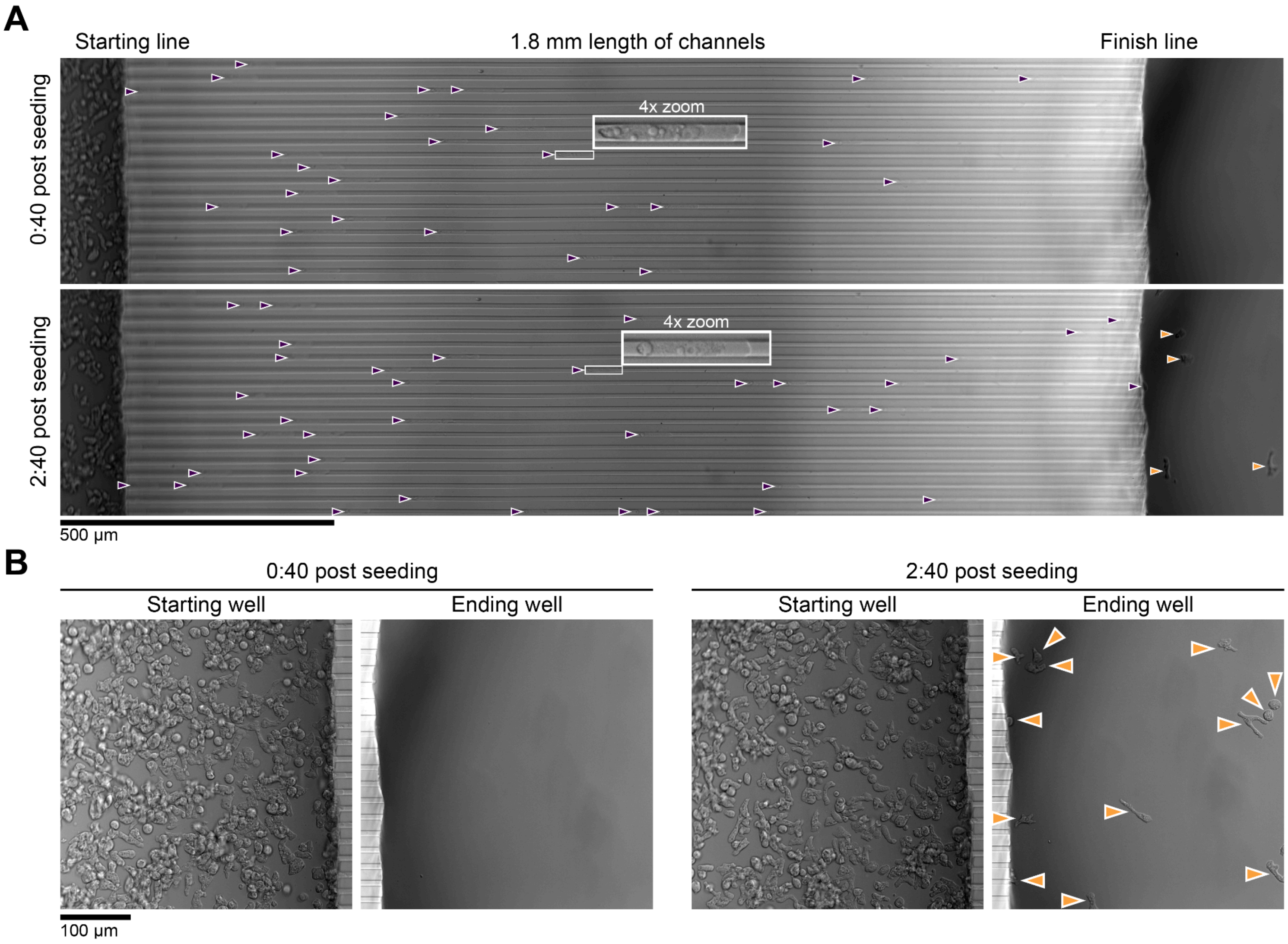

Figure S2. *Naegleria* amoebae can successfully complete a 1.8 mm dash. (A) *Naegleria* amoebae were seeded into a single well (left) of a dish with 8 µm channels, and imaged for 2 hours, starting 40 minutes post seeding. Images cover the full 1.8 mm run and the adjacent starting and ending wells. A single focal plane where the channels are in focus is shown for each timepoint. 4X insets show magnified amoebae from an optimized focal plane. Cells in channels are noted with purple arrowheads, cells that traversed the entire run length to reach the next well are shown with orange arrowheads. **(B)** Higher magnification views at optimized focal planes from the timepoints in A are shown for the starting well (where cells were seeded) and the ending well (where cells crawled). Cells that made it past the finish line are labeled with an orange arrowhead.

bioRxiv preprint doi: https://doi.org/10.1101/2025.11.30.691445; this version posted December 1, 2025. The copyright holder for this preprint (which was not certified by peer review) is the author/funder, who has granted bioRxiv a license to display the preprint in perpetuity. It is made available under aCC-BY 4.0 International license.

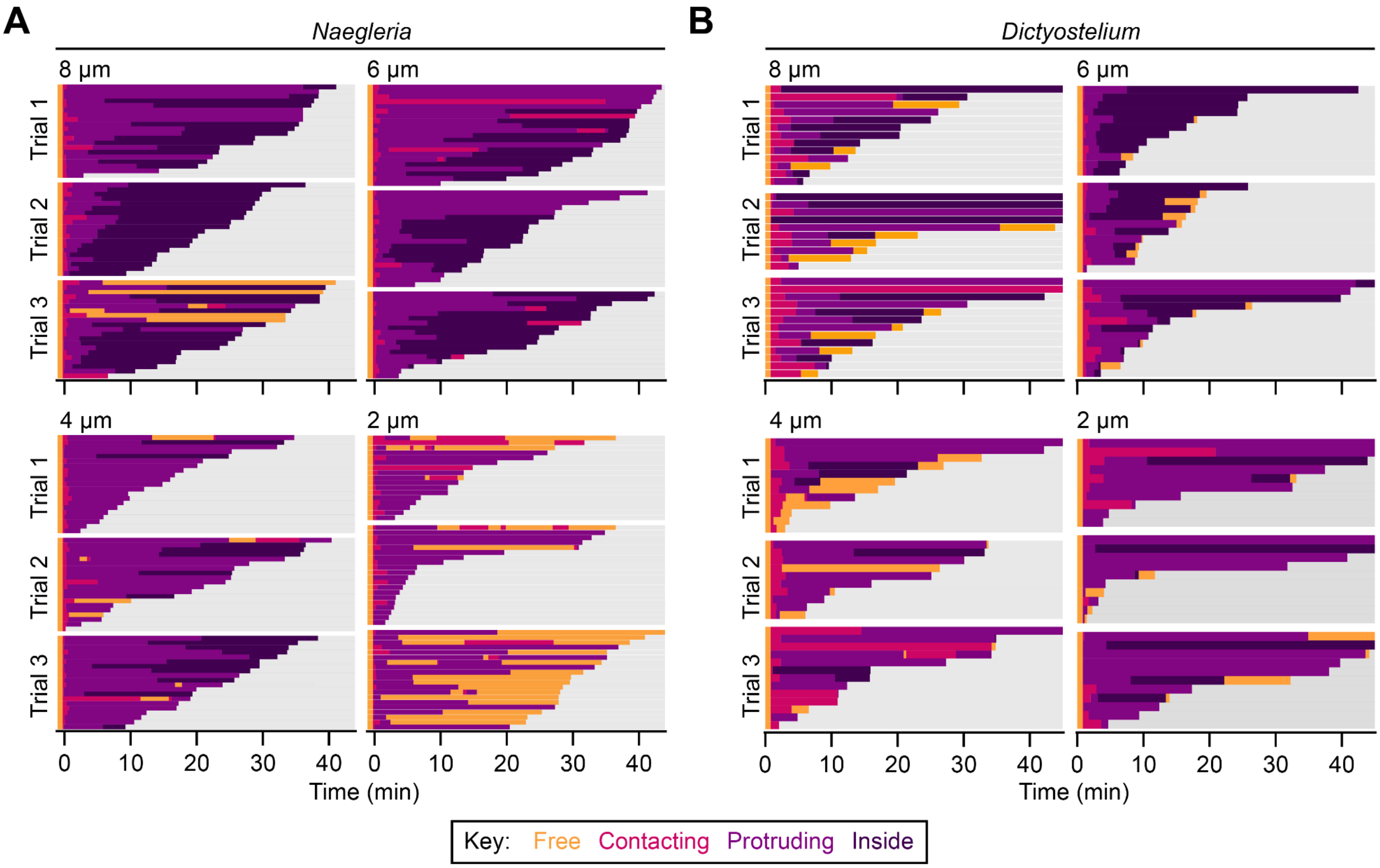

Figure S3. *Naegleria* enters microchannels at high rates. (A) *Naegleria*'s or **(B)** *Dictyostelium*'s interactions with 2-8 µm channels over 45 minutes of imaging are shown as horizontal bar plots, normalized such that t=0 represents the cell's first contact with the interface. Trial 1 for *Naegleria* is shown in Fig. 3B.

bioRxiv preprint doi: https://doi.org/10.1101/2025.11.30.691445; this version posted December 1, 2025. The copyright holder for this preprint (which was not certified by peer review) is the author/funder, who has granted bioRxiv a license to display the preprint in perpetuity. It is made available under aCC-BY 4.0 International license.

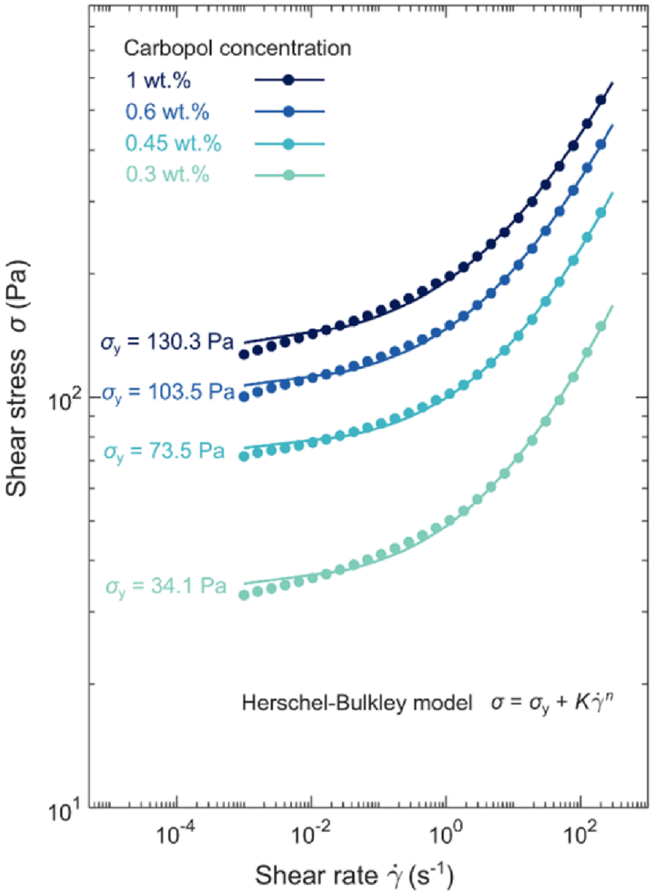

Figure S4. Rheological measurement of the Carbopol granular matrices. Shear stress of different Carbopol concentration versus unidirectional shear rates. The curves are fitted with the Herschel-Bulkley model for shear-thinning, viscoplastic fluids, where σ_y represents the yield stress, K is the consistency index, n is the flow index, and $\dot{\gamma}$ is the shear rate.
